# Prevalence of *Plasmodium falciparum* Infection in Rainy Season, Artibonite Valley, Haiti, 2006

**DOI:** 10.3201/eid1310.070567

**Published:** 2007-10

**Authors:** Thomas P. Eisele, Joseph Keating, Adam Bennett, Berlin Londono, Dawn Johnson, Christina Lafontant, Donald J. Krogstad

**Affiliations:** * Tulane University School of Public Health and Tropical Medicine, New Orleans, Louisiana, USA; † Hôpital Albert Schweitzer, Deschapelles, Haiti

**Keywords:** Malaria, prevalence, Haiti, community survey, dispatch

## Abstract

We conducted a population-based survey to estimate the prevalence of *Plasmodium falciparum* infection among persons older than 1 month in the Artibonite Valley of Haiti during the high malaria transmission season in 2006. Results from PCR for 714 persons showed a prevalence of 3.1% for *P. falciparum* infection.

Lying just 700 miles from the United States, Haiti is 1 of only 2 countries in the Caribbean with endemic transmission of *Plasmodium falciparum* malaria. Reportedly, 74%–80% of Haiti’s population live in malarious areas <300 m elevation (*1**,**2*). *Anopheles albimanus* has been identified as the vector responsible for nearly all malaria transmission in Haiti (*3*). However, reliable population-based estimates of the distribution and impact of malaria in Haiti are scarce (*4*); existing data are primarily from confirmed malaria cases reported through the health system. The seasonal peak in malaria transmission typically occurs from November through January, following the main rainy season (*5*). We conducted a population-based survey to estimate the prevalence of *P. falciparum* infection among persons older than 1 month in the Artibonite Valley of Haiti during the high transmission season in 2006.

## The Study

This research was conducted in the Artibonite Valley. Urban areas were excluded. This site was chosen because of its low altitude and abundant rainfall, as well as the large number of malaria cases historically seen at hospitals in the area (*5**–**7*). The Artibonite Valley is heavily farmed; 80% of the area is irrigated for cultivation of rice and other crops.

**Figure 1:**
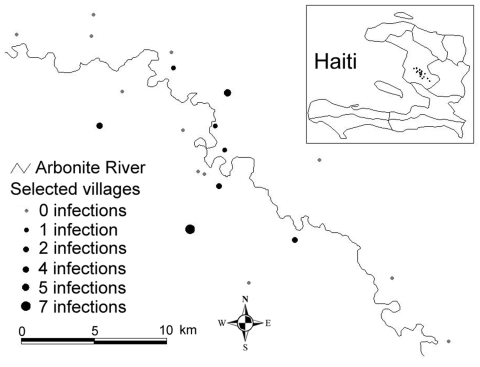
Map of selected villages for estimating the prevalence of *Plasmodium falciparum* infection, including number of infections identified within each village, Artibonite Valley, Haiti, 2006.

A 2-stage cluster design, probability proportional to cluster size, was used to generate a probability sample of 200 households within the study area; 20 primary sampling units were selected at the first stage and 10 households at the second stage (Figure 1). Fieldwork was conducted by trained data collectors from November 20 to December 10, 2006. Ethical approval was obtained from Tulane University and Hôpital Albert Schweitzer (HAS).

After informed consent was obtained, blood samples were collected and axillary temperature determined for all persons older than 1 month within each selected household. Thick and thin blood films were prepared for each person, as well as 4 blots of blood on filter paper for PCR. Up to 3 return visits were made to each household to limit nonresponse. Basic personal and household demographic data were collected through an interview with the designated head of household; a standardized questionnaire was used for all 200 households eligible for inclusion in the survey.

Using standard methods (*8*), a trained laboratory technician at HAS interpreted the malaria blood slides. Positive persons were treated with chloroquine. Filter paper blots were transported back to the laboratory at Tulane University for PCR analysis to test for *P. falciparum* parasites. Blood samples on filter paper from microscopy-confirmed infections and respondents with temperatures >37.5°C were analyzed individually by PCR for *P. falciparum*. Pooled PCR analysis of 10 samples was used to detect *P. falciparum* infections in filter paper samples from respondents with negative microscopy results; positive pools were then analyzed individually.

Positive specimens were identified on the basis of PCR for conserved sequences in 18S small subunit RNA, with a single reverse primer for all *Plasmodium* species and a *P. falciparum–*specific forward primer (*9*); expected amplicon size was 276 bp for *P. falciparum.* The positive control contained DNA from a culture of the *P. falciparum* Haiti strain. The negative control contained water instead of DNA. The amplified electrophoresis products were visualized on a 1% agarose gel and stained with ethidium bromide.

Prevalence of *P. falciparum* infection was calculated as the proportion of sampled persons with a positive PCR result divided by the number of persons who provided blood samples. All point estimates were weighted, with empirically estimated standard errors used to account for clustering.

A total of 804 persons older than 1 month were eligible for inclusion in the survey; 714 agreed to provide a blood sample. This resulted in a nonresponse rate of 11.2% for estimating malaria parasite prevalence. Ages of persons in the sample ranged from <1 to 92 years; 46.1% were male. Ninety-one children <5 years of age (12.7%) were included in the sample. A total of 8.6% of the persons were considered febrile (axillary temperature >37.5°C).

Microscopy at HAS identified 7 malaria infections among the 714 persons who had provided a blood sample; all were confirmed by PCR. Diagnosis by individual and pooled PCR of the remaining blood samples on filter paper identified an additional 16 *P. falciparum* infections, totaling 23. Thus the total prevalence was estimated to be 3.1% (95% confidence interval 0.60%–5.7%) (Table). The resulting sensitivity and specificity of microscopy were 30.5% and 100%, respectively. Persons with infections ranged in age from 1 to 62 years; 65.2% were male. A total of 14.2% of febrile persons had positive malaria results, compared with only 2.1% of nonfebrile persons. Of the 20 villages included in the sample, all 23 persons with malaria infections came from 8 villages, which were 26–319 m above sea level.

**Table:** Malaria parasite prevalence by demographic characteristics, Artibonite Valley, Haiti, 2006

Characteristic	No. malaria infections identified*	No. respondents tested	Parasite prevalence, %†
Age, y			
<5	2	91	2.2
5–9	5	100	4.8
10–19	2	186	1.5
20–29	6	95	4.7
30–39	2	59	3.8
40–49	3	69	4.0
50–59	1	42	5.1
>60	1	50	1.6
Unknown	1	22	3.5
Sex			
Male	15	329	3.9
Female	8	385	2.3
Temperature			
Febrile (>37.5°C)‡	9	61	14.2
Nonfebrile	14	647	2.1
Total	23	714	3.1§

*Plasmodium falciparum infections only. Results based on PCR, which includes all microscopy-confirmed infections. †Prevalence point estimates are weighted. ‡Sample size for febrile versus nonfebrile; 6 missing data records. §95% confidence interval 0.6–5.7.

## Conclusions

Results from the survey show the prevalence of *P. falciparum* infection to be 3.1% in this area of Haiti. To our knowledge, this is the first population-based estimate of malaria parasite prevalence in Haiti that used PCR diagnosis. Among febrile persons, whose prevalence was 14.2%, our results are substantially higher than previous estimates from passive surveillance of suspected malaria case-patients (*5*,*6*,*10*).

**Figure 2:**
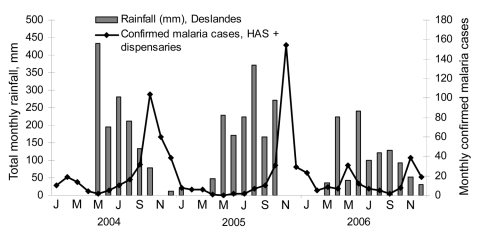
Microscopy-confirmed malaria cases at Hôpital Albert Schweitzer (HAS) and total monthly rainfall, 2004–2006, Deslandes, Artibonite Valley, Haiti.

While moderate, a 3.1% prevalence represents a substantial level of illness, especially when one considers that the severity of the disease is likely high given the low level of acquired immunity among the Haitian population. Furthermore, based on passive case detection of confirmed malaria cases identified by HAS in the Artibonite Valley from 2004–2006 (Figure 2), transmission in 2006 appears to have been substantially lower than in previous years. Thus the population-based prevalence estimate of 3.1% likely represents the lower bound of the malaria impact in this area.

Malaria transmission was highly localized; all 23 infections were in persons from 8 villages (40%), which suggests that transmission is potentially based on a set of discrete ecologic determinants (Figure 1). Such clustering is consistent with the observed tendency for *Anopheles* mosquitoes to overdisperse (*11*). Surprisingly, 7 (30%) of the 23 infections were in persons from a village 319 m above sea level, although the exact location of inoculation cannot be confirmed. If transmission occurred at this elevation, it is above what has commonly been understood as the upper bound for transmission in Haiti.

The observed low level of sensitivity of microscopy compared with that of PCR for identifying *P. falciparum* infections is similar to findings observed elsewhere (*12**–**14*). We surmise that such a low level of sensitivity was attributable to 2 factors: 1) many of the infections likely occurred at low parasite densities, and 2) the laboratory technician was responsible for reading a large number of slides with low parasite prevalence over a relatively short period.

We argue that future malaria interventions in Haiti should be directed toward controlling malaria in the context of a moderate transmission setting; thus, large-scale distribution of insecticide-treated nets or widespread use of indoor residual spraying may be less cost-effective than enhanced surveillance with effective case management or focused larval control. A key aspect of future research in Haiti should therefore focus on understanding treatment-seeking behavior, barriers to accessing health services among febrile persons, and quantifying patterns of malaria transmission.
